# Ultra stable, inkjet-printed pseudo reference electrodes for lab-on-chip integrated electrochemical biosensors

**DOI:** 10.1038/s41598-020-74340-1

**Published:** 2020-10-13

**Authors:** Sotirios Papamatthaiou, Uros Zupancic, Curran Kalha, Anna Regoutz, Pedro Estrela, Despina Moschou

**Affiliations:** 1 Centre for Biosensors, Bioelectronics and Biodevices (C3Bio) and Department of Electronic and Electrical Engineering, University of Bath, Bath, BA2 7AY UK; 2 Department of Chemistry, University College London, London, WC1H 0AJ UK

**Keywords:** Biochemistry, Chemistry, Engineering, Materials science

## Abstract

Lab-on-Chip technology comprises one of the most promising technologies enabling the widespread adoption of Point-of-Care testing in routine clinical practice. However, until now advances in Lab-on-Chip have not been translated to the anticipated degree to commercialized tools, with integrated device mass manufacturing cost still not at a competitive level for several key clinical applications. Lab-on-PCB is currently considered as a candidate technology addressing this issue, owing to its intuitive compatibility with electronics, seamless integration of electrochemical biosensors and the extensive experience regarding industrial manufacturing processes. Inkjet-printing in particular is a compatible fabrication method, widening the range of electronic materials available and thus enabling seamlessly integrated ultrasensitive electronic detection. To this end, in this work stable pseudo-reference electrodes are fabricated for the first time by means of commercial inkjet-printing on a PCB-integrated electrochemical biosensing platform. SEM and XPS analysis are employed to characterize the electrodes’ structure and composition and identify any special characteristics, compared to published work on alternative substrates. Additionally, this paper analyzes integrated reference electrodes from a new perspective, focusing mainly on their characteristics in real-life operation: chemical sintering as opposed to high budget thermal one, stability under continuous flow, pH dependency and bias stress effects on electrode instability, a parameter often overlooked in electrochemical biosensors.

## Introduction

In the past few years, significant progress in Lab-on-Chip (LoC) technology for diagnostic devices has been reported^1–3^. Even though there have been some commercialization examples of LoC devices^4^, few products have managed to achieve widespread commercial use. A major reason for this is the lack of standardized design and manufacturing processes for LoC commercial upscaling, which results in an increased cost for such integrated devices^5,6^.

Even though a range of various materials (e.g. silicon, glass, paper, PDMS) have been investigated for Lab-on-Chip devices^6–9^, they all still face the same aforementioned commercial upscaling bottleneck^5^. To this end, the printed circuit board (PCB) industry has emerged as a promising platform that could support the commercialization of complex, integrated Lab-on-Chip devices, establishing the Lab-on-PCB approach as a unique candidate for standardized LoC development across industry and academia^5^. The low cost of the devices due to economy of scale manufacturing, along with the intuitive electronics and electrochemical biosensor integration are Lab-on-PCB’s unique features that can drive the LoC concept to achieving its anticipated potential and real-life impact.

Additionally, inkjet-printing has recently evolved into a favorable deposition method in PCB manufacturing^10^, leading in a merging of the fields of PCBs and inkjet printed electronics, with numerous PCB factories incorporating printing capabilities as a standard in their facilities. This progress has been boosted by developments in monitoring and simulations of advanced, functional ink rheology and surface tension in the droplet formation^11^. The effective matching of ink properties to the specifications of the employed printhead makes the printing of a wide range of novel materials on a variety of substrates feasible. Inkjet-printing seems to have all the characteristics (i.e. maskless, drop-on-demand, large scale upscalability) to qualify as the ideal deposition method for advanced functional material deposition with respect to Lab-on-PCB applications.

Even though there are previously published studies utilizing PCB reference electrodes^12,13^ or inkjet-printed reference electrodes^14–16^, none of them have incorporated inkjet-printed Ag/AgCl reference electrodes on commercially manufactured PCB electrochemical biosensing platforms. Herein, we present for the first time ultra-stable inkjet-printed Ag/AgCl pseudo-reference electrodes on PCB substrates, without the need of any additional coating. This architecture enables the use of integrated reference electrodes (Ag/AgCl being the standard type)^17^ on standard gold-plated working and counter microelectrode biosensing configurations, avoiding the need for a laborious deposition of Ag on the existing gold-plated substrate, or the requirement for an additional Ag plated layer.

Evaluation of the electrode electrical stability under static and flow condition for various post-printing treatments and pH dependence is described within this work, showing that our chemically sintered pseudo-reference electrodes are the most stable inkjet-printed ones in the literature so far, and thus are fit to be used in real-life sensing applications with increased repeatability and reliability requirements under continuous flow of reagents. Finally, the reference electrode aging under DC voltage bias stress is evaluated, quantifying the importance of reference electrode drift after continuous biasing cycles; the proposed stress process is the first one ever proposed to assimilate the real-life operation of such electrodes in electrochemical biosensing experiments.

## Experimental

**Figure 1:**
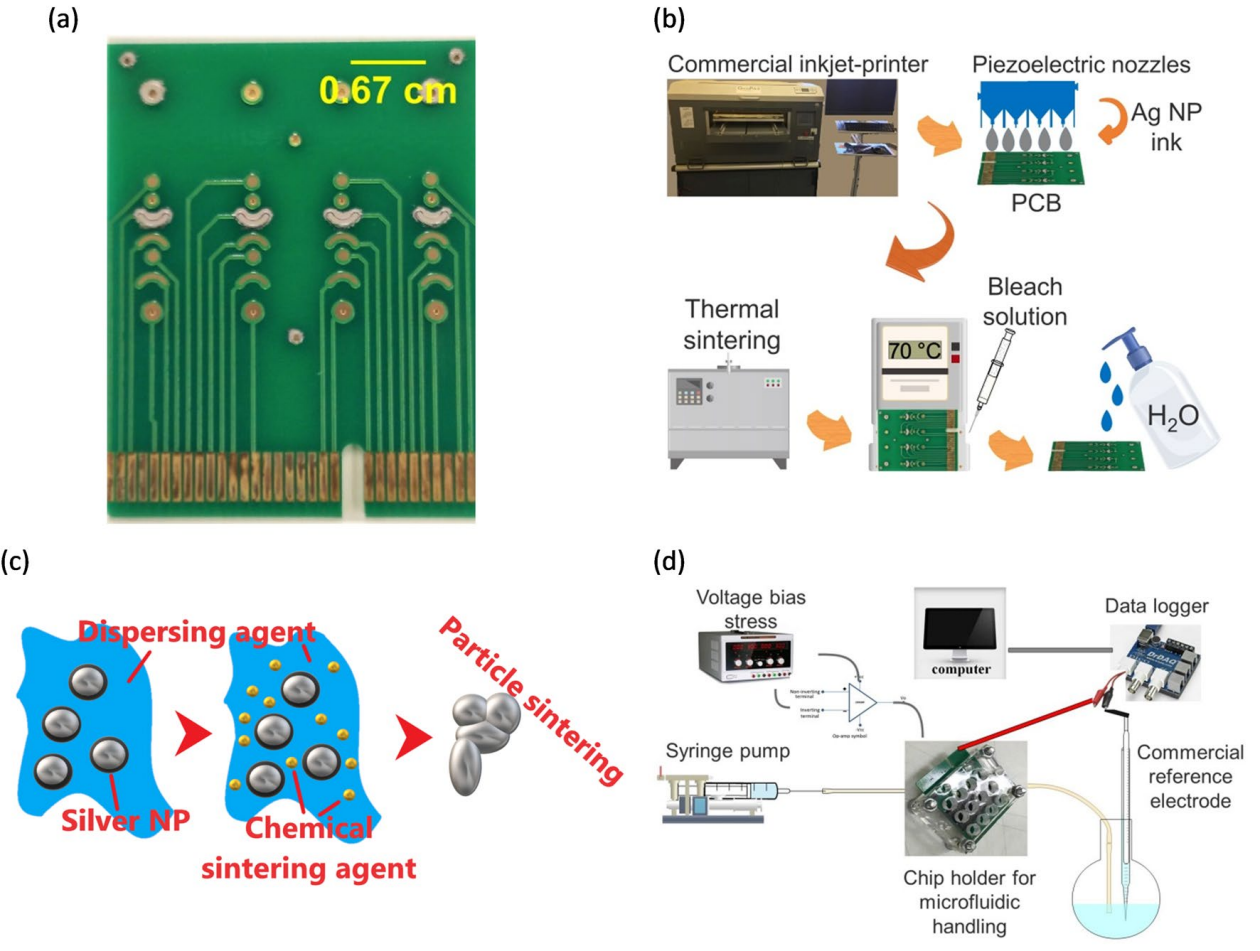
(**a**) Photograph of the PCB with four thermally-sintered Ag electrodes, inkjet-printed on gold, (**b**) generic schematic of the reference electrode fabrication process, (**c**) schematic of Ag NP ink chemical sintering, (**d**) schematic of the measurement setup.

The prototype PCB platform, which was used to study the printed pseudo-reference electrodes (PRE), was designed in Altium and manufactured with commercially available PCB technologies by Lyncolec Ltd, UK^18^. The board dimensions are 3.5 cm × 4.2 cm with four columns of gold electrodes of various geometrical shapes (Fig. 1a). A pair of inlet/outlet vias is drilled on each column to facilitate subsequent microfluidic channel integration. The PCBs were cleaned prior to printing in a sonication bath for 15 min in acetone, 15 min in ethanol and 30 min in a mixture of 1 (NH_4_OH) : 1 (H_2_O_2_) : 5 (H_2_O). Immediately before printing, the PCBs were further cleaned with a UV/ozone cleaner for 40 min (20 min UV exposure and 20 min in the ozone, ProCleanerTM BioForce Nanosciences).

A large format, commercial inkjet-printer (CircaPrint 6080, Viking Test Ltd UK) was used to print a commercial silver nano-particle ink (Mitsubishi Paper Mills Limited) on top of the gold electrodes. A schematic of the reference electrode fabrication process is depicted in Fig. 1b. The used Ag ink is water-based with 20 nm silver nanoparticles (1.26 g/mL) and a viscosity of 4 cP at 25 °C with a surface tension of 32 mN/m. A transferable hot plate was installed on the printing table to elevate the substrate temperature during printing. The printable designs were prepared in Altium, exported as Gerber files and imported into the CircaPrint software. The camera aided alignment features were used to print the patterns precisely on the PCB boards. After a brief initial evaluation of the printing parameters controlled by the printer, including the droplet size, the resolution and the uni- or bi-directional printing, optimum parameters were selected (minimum droplet size: 1.5 pL, maximum resolution: 1440 dpi and unidirectional printing). The printing processes were carried out in a standard laboratory environment under ambient conditions without temperature or humidity control. This was favored to investigate the viability of mass production following the standard industrial practice.

Material sintering enables the removal of various chemical species, including surfactants that are bound to the metallic nanoparticles. These species are responsible for the stability of the metallic nanoparticles in ink form and the separation enables particle fusion, thus electron transfer. Thermal and chemical treatments are well-established sintering methods for silver nanoparticle inks^11,14^. Chemical compounds of chlorine (Cl) are usually used for chemical sintering where the stabilizing layer is detached from the nanoparticles letting them to coalescence (Fig. 1c)^11,19,20^. In this work, two sintering procedures were followed and compared regarding their PRE stability: thermal sintering at 160 °C in furnace for 1 h and chemical sintering by placing the electrode on a hot plate at 70 °C and drop-casting 10 μL of 0.5% (v/v) HCl in H_2_O. After the ink sintering, silver chlorination was achieved by drop-casting 10 μL of sodium hypochlorite solution (6–14% active chlorine) to produce the AgCl layer, necessary to fabricate Ag/AgCl PRE^13^. The deposition lasted for 1 min and then the electrode was washed with de-ionised water. A change in color was observed after this stage from silver to dark grey, visually indicating surface chlorination^13,21^.

Scanning Electron Microscopy (SEM) and X-ray Photoelectron Spectroscopy (XPS) were used to characterize the surface of the printed electrodes. SEM analysis was conducted using a JEOL JSM-6480LV Tungsten filament SEM, using a 10 kV accelerating voltage. XPS was performed on a Thermo Scientific K-Alpha + XPS system, which uses a monochromated, microfocused Al Kα X-ray source (hν = 1486.7 eV) and a 1800 double focusing hemispherical analyzer with a 2D detector. Data were collected at 200 eV pass energy for survey and 50 eV pass energy for core level spectra using an X-ray spot size of 400 μm. Samples were mounted on conducting carbon tape and a flood gun was used to minimize sample charging. All qualitative and quantitative data analysis was performed using the Avantage software package. Core level spectra are normalized to the combined peak area of the Ag 3d_5/2_ and Cl 2p core levels to aid comparison.

The stability of the PRE was assessed with Open Circuit-Potential (OCP) measurements versus commercial Ag/AgCl (KCl) reference electrode (BASi) in buffer solutions, AVS Titrinorm (pH 4, pH 7, pH 10, VWR Chemicals). The electrical measurement setup is depicted in Fig. 1d. A data logger (DrDAQ, Pico Technology) was used to measure the potential difference (V_PCB_ − V_commercial_). A standard Peripheral Component Interconnect (PCI) board connector is used to electrically connect the PRE with the data logger. Initially, non-flow measurements were performed to optimize the printing parameters, followed by measurements under flow for 24 h. A microfluidic delivery network was laser micromachined and attached on the PCB via pressure-sensitive adhesive tape^18^. The pH buffer was flowed through the channel by a syringe pump (Cole-Parmer, USA) at a constant flow rate (7 μL/min). The remarkably consistent behavior between different electrodes (minimum four electrodes of various printing and material treatment parameters were studied) allowed the demonstration of representative curves.

Finally, the DC voltage bias stress influence on the PRE stability was assessed. This was experimentally investigated by measuring the OCP under flow for 20 min before and after 1 min of continuous voltage bias. Three consecutive biases of 0.3 V were applied, with the same procedure followed for 0.6 V and 0.9 V biases. These voltage levels are within the range of common gate electrode biases for electrolyte gated transistors^22–26^. A high input impedance operational amplifier was operated at unity mode and was connected between the voltage source unit and the PRE to inhibit any current flow to the latter. Thus, the operation of a gate reference electrode of an electrolyte gated transistor can be efficiently simulated and evaluated.

## Results and discussion

### Material characterization

**Figure 2:**
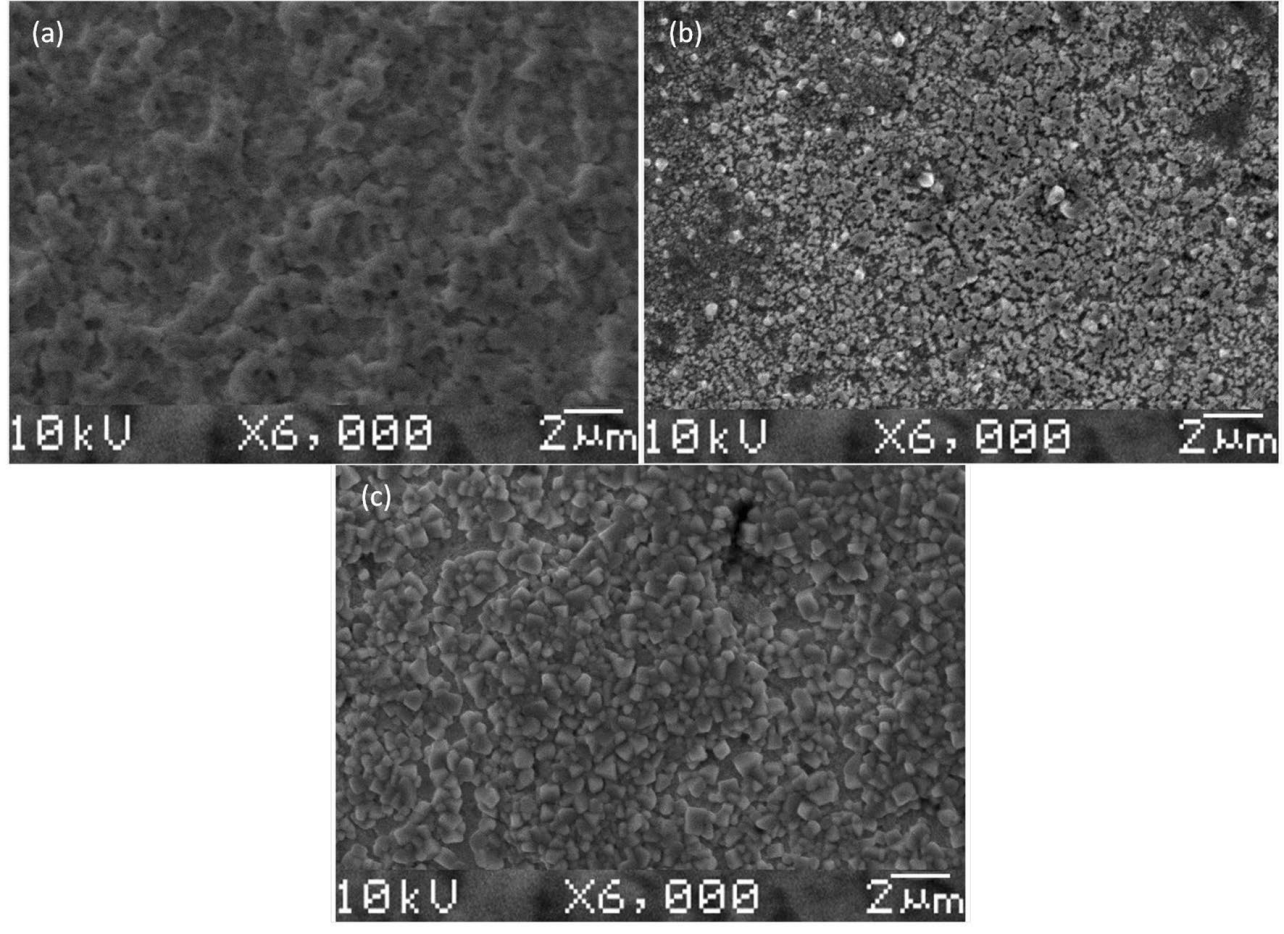
SEM images of inkjet-printed silver nano-particle ink (**a**) thermally sintered at 160 °C for 1 h, (**b**) chemically sintered by drop-casting 10 μl of 0.5% (v/v) HCl in H_2_O and (**c**) chlorinated with sodium hypochlorite solution (6–14% active chlorine).

SEM analysis was employed to examine the surface morphology of each of the inkjet-printed Ag layers. Figure 2 shows that the surface of the thermally sintered sample (Fig. 2a) is relatively continuous with neck formation whereas a development of individual particles (approximately 300 nm diameters; ImageJ 1.52a software was used for quantitative feature extraction) can be observed on the chemically sintered sample (Fig. 2b). Following sodium hypochlorite treatment (Fig. 2c), this pattern is further intensified and larger particles (more than 650 nm diameters) become apparent. The particle formation for chemical sintering and sodium hypochlorite treatment can be attributed to the swelling of the silver nano-particles due to the formation of silver chloride layers, as reported also by Moschou et al.^13^ da Silva et al.^15^ and Qin et al.^16^.

**Figure 3:**
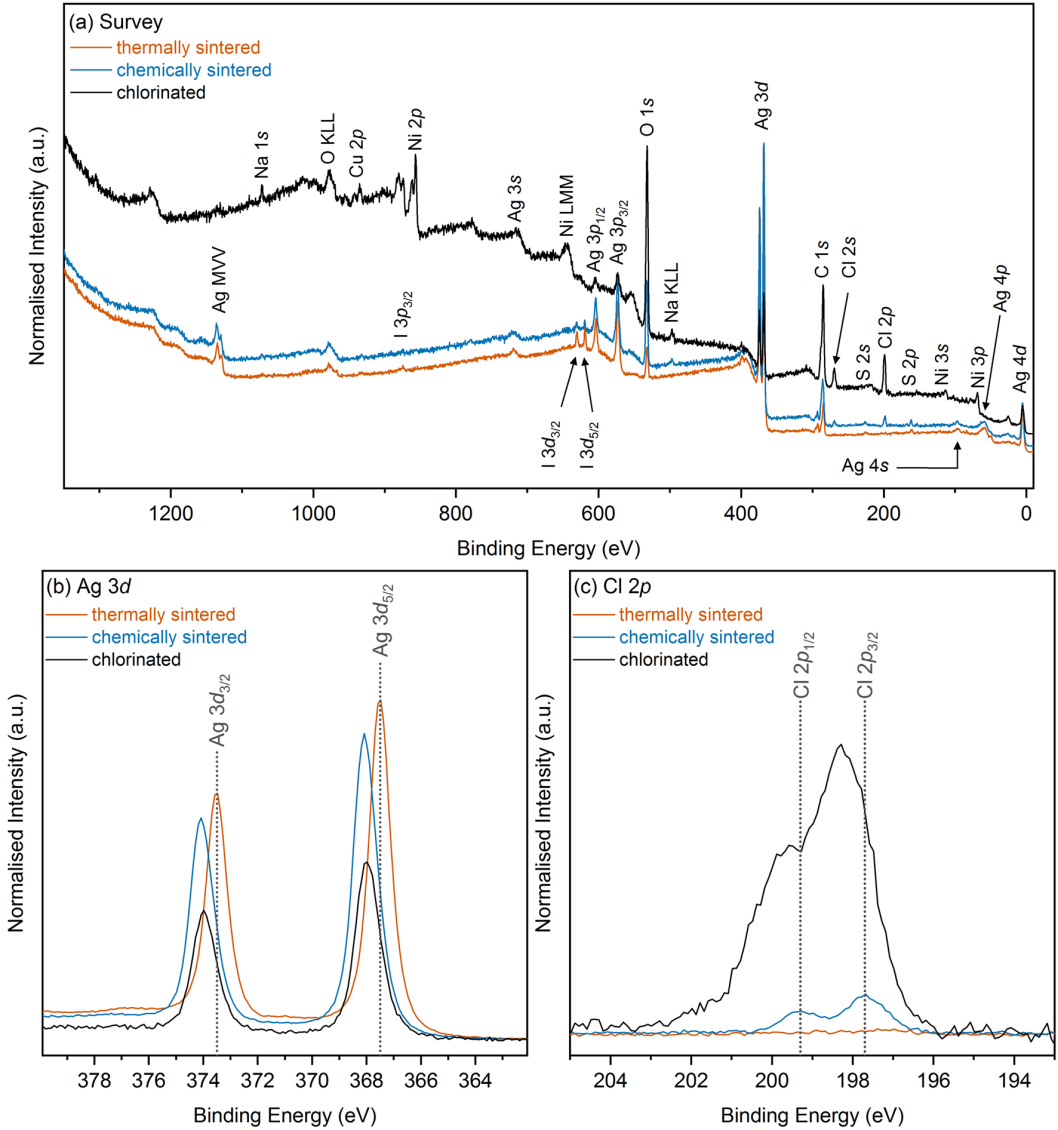
XP spectra of thermally and chemically sintered as well as chlorinated electrodes. (**a**) Survey spectra with all core levels and Auger lines indicated, (**b**) Ag 3d and (**c**) Cl 2p core level spectra.

XPS was performed to investigate the surface chemistry of the printed electrodes after the various treatments. Figure 3a shows the survey spectra including all major core and Auger lines. The thermally and chemically sintered electrodes show strong Ag signals, with additional contributions from Cl, K, I, C, and O. C and O are mainly due to surface contamination layers, which are easily visible due to the surface sensitive nature of XPS. I and K are attributed to the proprietary ink composition of Mitsubishi^27^. The chlorinated sample shows a more complex spectrum including additional metal signals from Ni and Cu, as anticipated by exposure to the underlying layers of the PCB (Cu metal layer with electrodeposited Ni as an adhesive layer for the final gold plating). Figure 3b,c show the Ag and Cl core levels of the investigated electrodes. The Ag 3d core level occurs at binding energies (BE) typical for metallic Ag and AgCl. These two chemical environments are within 0.1 eV from each other and are therefore not resolved as individual peaks. The BE shifts observed are due to a change in the relative contributions from these two environments as changes in relative intensities of the two components are detected as an overall shift in BE. In addition, a contribution from Ag_2_O is observed as a small shoulder at lower BE to the main core line peak in the chemically sintered and chlorinated samples. The thermally sintered sample only shows trace amounts of Cl (3.6 rel. at% compared to Ag as determined from total peak area analysis of the XPS core level data), whilst the chemically sintered and chlorinated samples show the expected double peak with Cl levels of 17.6 and 76.3 rel. at% compared to Ag. Table S1 in Supplementary Information summarizes all relative atomic ratios from peak fits to the core level spectra and Figure S1 (Supplementary Information) shows the remaining core level spectra.

### Non-flow OCP characterization

**Figure 4:**
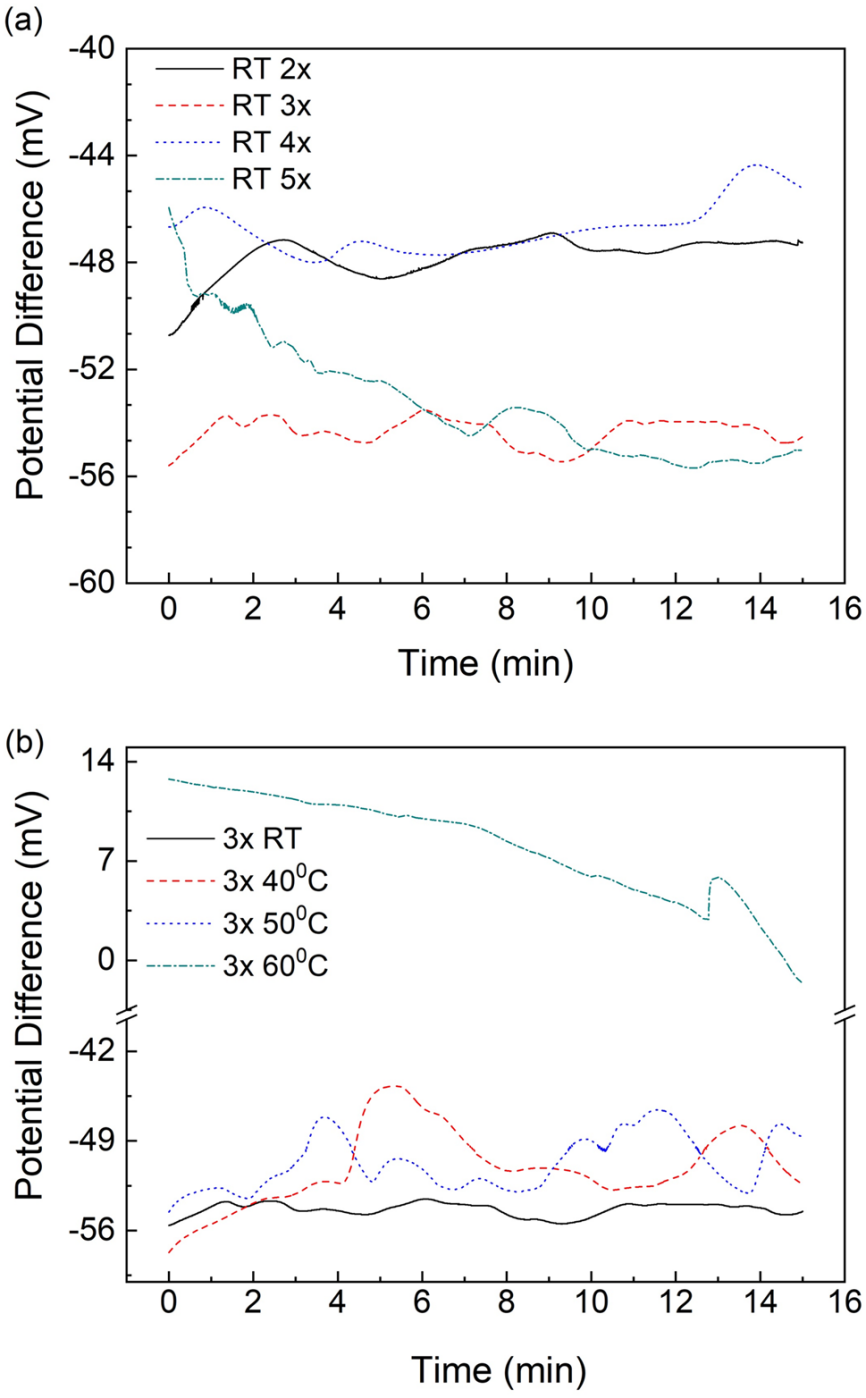
The potential difference between the commercial Ag/AgCl (KCl) reference electrode and the inkjet-printed thermally sintered electrodes in pH 7 buffer for (**a**) various printing cycles at room temperature and (**b**) various printing temperatures at three printing cycles.

Non-flow characterization of the PRE was initially performed in order to define the optimal fabrication parameters. The stability of the thermally sintered silver PRE was assessed by immersion in a pH 7 buffer solution in static conditions for 15 min. Room temperature (RT) printing and 3 printing cycles were eventually selected as this exhibited the most stable behavior among 40 °C, 50 °C and 60 °C printing temperatures and 2, 4 and 5 printing cycles (Fig. 4). A pronounced drift is observed for 5 printing cycles whereas 2, 3 and 4 cycles exhibit more stable performance (Fig. 4a). Figure 4b shows the potential difference dependency on the printing temperature. Elevated printing temperatures lead to unstable behavior with potential fluctuations that are characterized by an intensified drift for the higher tested printing temperature (60 °C). The drift values for every sample are summarized in Table 1. The inferior behavior of samples after 2 printing cycles and the elevated temperature prints was attributed to partial surface coverage, which leaves the underlying gold exposed, whereas 4 and 5 printing cycles resulted in a thicker material (ink overflow) making it susceptible to deformations and washouts.

**Table 1:** OCP drift values of inkjet-printed thermally sintered electrodes in pH 7 buffer for different printing parameters.

Printing cycles at RT	Drift (mV/h)	Printing temperature (°C) of 3 ×	Drift (mV/h)
2 ×	14.08	RT	4.16
3 ×	4.16	40	21.48
4 ×	6.44	50	23.44
5 ×	36.08	60	57.56

**Figure 5:**
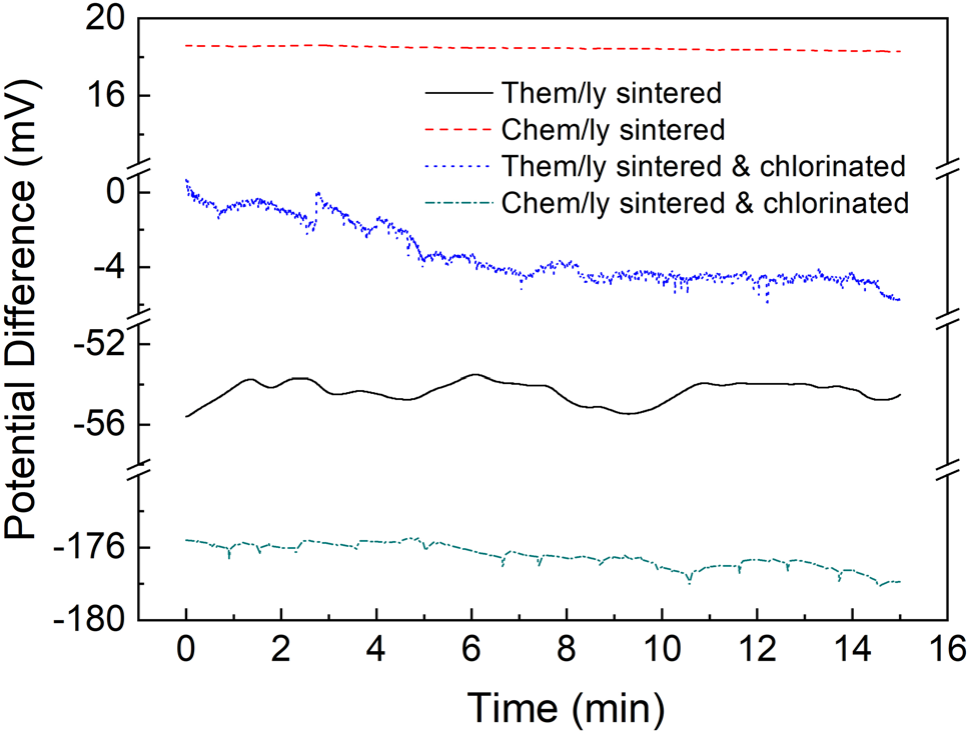
The potential difference between the commercial Ag/AgCl (KCl) reference electrode and the PREs in pH 7 buffer for thermally and chemically sintered PREs and their chlorinated counterparts.

Subsequently, we proceeded with identifying the optimum sintering conditions through a comparison between thermally and chemically sintered PREs and their chlorinated counterparts (Fig. 5). It is evident that the chemically sintered PRE is the most stable material with no potential fluctuations. Compared to the thermally sintered, it features a ten times lower standard deviation from the mean value (0.06 mV vs. 0.6 mV). Both chlorinated samples show a clear drift of 20 mV/h for the thermally sintered & chlorinated and 9.2 mV/h for the chemically sintered & chlorinated.

### OCP characterization under continuous flow

**Figure 6:**
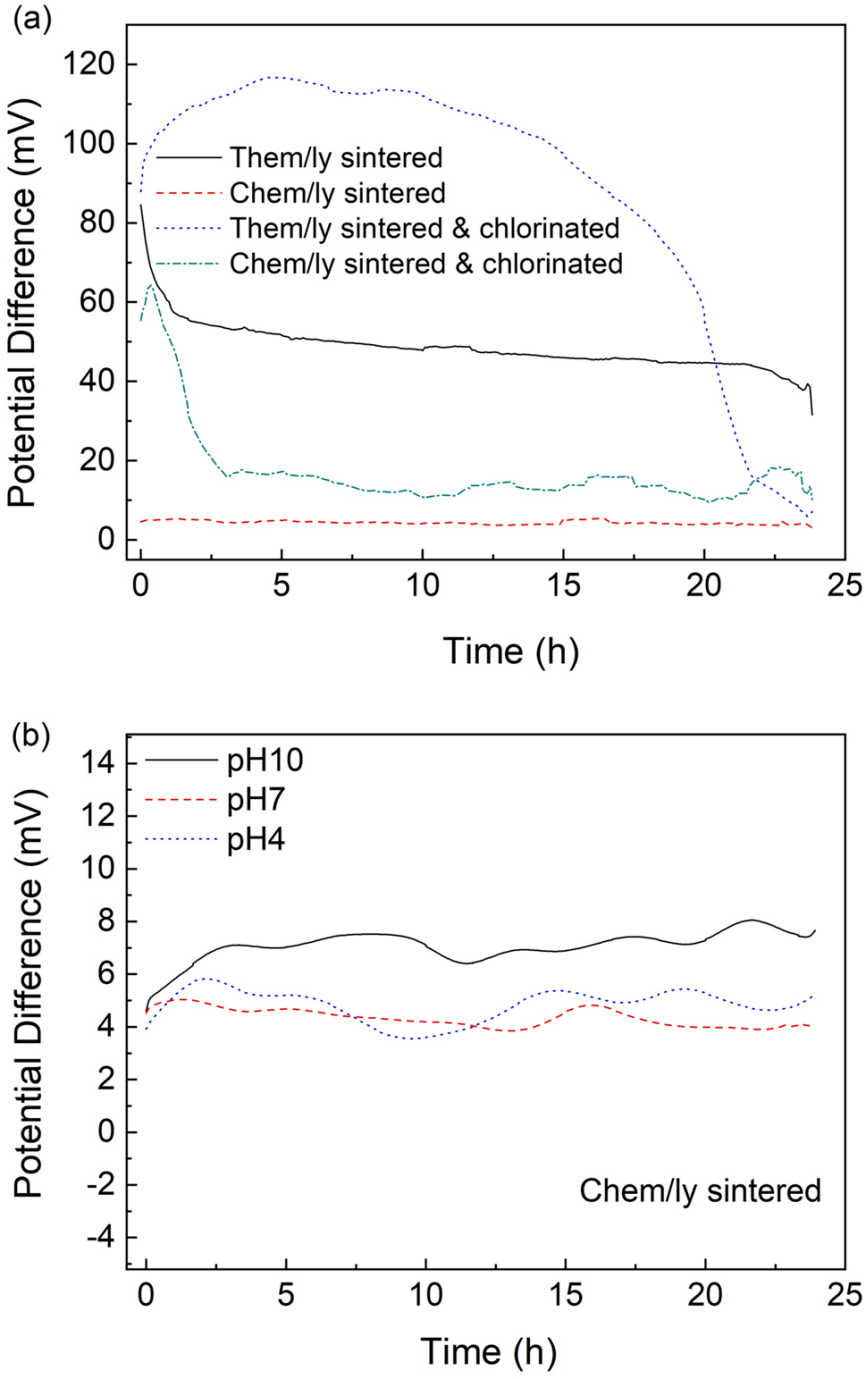
The potential difference between the commercial Ag/AgCl (KCl) reference electrode and the PREs under 7 μL/min flow of (**a**) pH 7 buffer for thermally, chemically sintered and their chlorinated counterparts and of (**b**) pH 10, pH 7 and pH 4 buffer for chemically sintered.

After having confirmed promising results regarding PRE stability compared to the commercial reference electrodes, OCP measurements under buffer flow were performed, considering the integration of such structures in real-life biosensing platforms where the μL-scale sample needs to be continuously injected over the electrodes. Figure 6a shows the OCP of the four different materials against the commercial reference electrode for a continuous 24 h flow of pH 7 buffer. It can be observed that the stability of the chemically sintered PRE is outstanding (0.04 mV/h), compared to reported inkjet-printed reference electrodes on a variety of substrates (Table 2).

**Table 2:** Comparison of inkjet-printed pseudo-reference electrodes with respect to reported drift values compared to commercial Ag/AgCl reference electrode.

References	Drift (mV/h)	Substrate	Printer	Ink
Moya^14^	0.13 (static, 24 h)	PEN	DMP 2831	AgNP (ANP Korea)
Da Silva^15^	No value reported, brief exposure to solution (static, < 30 min)	PET, paper	DMP 2800	AgNP (Sigma-Aldrich)
Qin^16^	0.6/0.3 (static, 16 h)	Glass/PI	DMP 2831	Non-commercial
This work	0.04 (flow, 24 h)	PCB	Circaprint 6080	AgNP (Mitsubishi Paper Mills Ltd)

Conversely, the thermally sintered silver electrode exhibited a drift (2 mV/h) as anticipated by their non-flow performance. The chlorinated electrodes also exhibited a significant drift of 4.5 mV/h for the thermally sintered & chlorinated and 1.9 mV/h for the chemically sintered & chlorinated electrodes. The poor performance of the chlorinated samples compared to the chemically sintered one could be attributed to the severe chlorination which consumed a significant percentage of silver volume and led to weak attachment to the underlying gold. This is supported by the fact that exposed gold was evident after the 24 h buffer flow whereas the non-chlorinated samples remained physically intact (Figure S2 in Supplementary Information). Moreover, tubing blockages at the outlet of the flow cell were frequent for the chlorinated samples, ascribed to the material delamination. Similar behavior was reported by da Silva et al.^15^ for silver that experienced increased reaction time with the chlorination agent. Further study of the chlorination process followed by reducing the reaction time to 2 s and decreasing the sodium hypochlorite concentration by 50%, but the electrode stability did not improve significantly.

Finally, the stability of the chemically sintered PREs was assessed under acidic (pH 4) and basic (pH 10) buffer flow (Fig. 6b). Apart from the impressive stability under flow for all three pH buffers, the potential difference to the commercial electrode is similar for every case, rendering the electrode virtually insensitive to pH changes within the 4–10 pH range and thus stable across a broad range of biological samples. It is worth mentioning that the chemically sintered electrode does not require any initial set-up time for the OCP to stabilize or any pre-conditioning. This is an attractive characteristic especially for Point-of-Care rapid diagnostic applications where the duration of the measurement is a significant parameter.

### Influence of DC voltage bias on PRE long-term operation stability

The influence of the DC voltage bias was evaluated on the PRE that demonstrated the best stability on the previous measurements (i.e. the chemically sintered one). This highlights the importance of stable pseudo reference electrodes under voltage bias application facilitating them as reliable gate electrodes of electrolyte gated transistors^23,25^. Employing this biosensing configuration in long-term measurements implies repetitive on/off bias cycles over long periods of time under continuous flow of reagents; the effect this pattern has on the reliability of the biosensor reading has not been probed until now.

**Figure 7:**
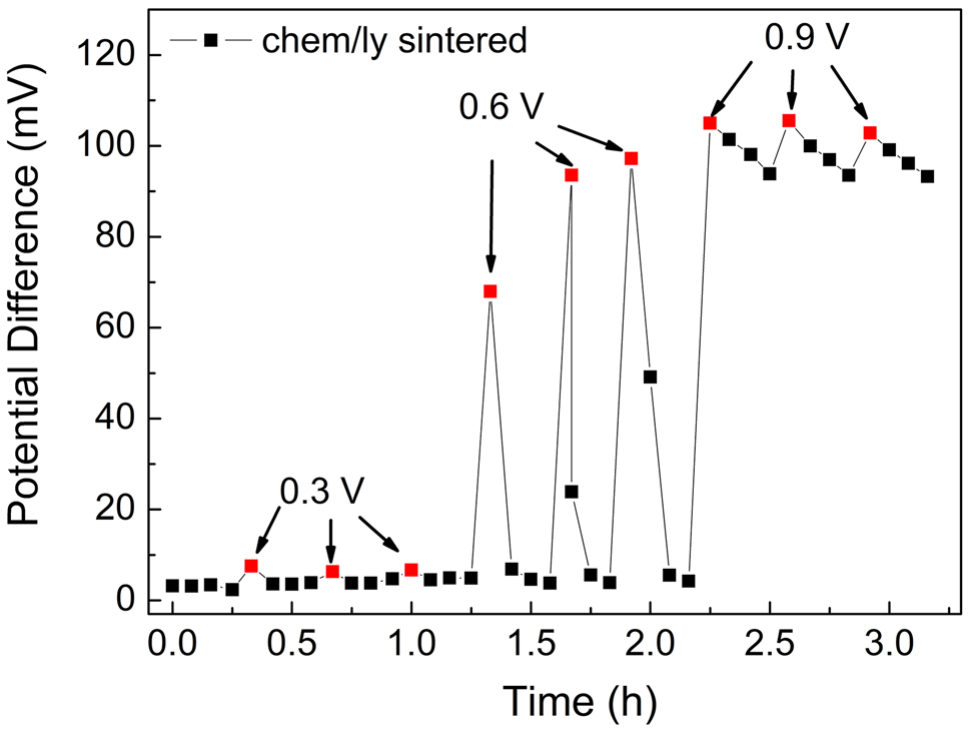
Influence of voltage bias stress on the stability of a chemically sintered PRE under 7 μL/min flow of pH 7 buffer. Each voltage bias level (0.3 V, 0.6 V and 0.9 V) was applied for three consecutive times (duration of each voltage bias was 1 min). The red dots are the first OCP values after each voltage bias.

Three different behaviors were observed: unstable behavior after the voltage bias where the signal did not recover the initial OCP value (before the voltage bias), stable behavior where the voltage bias did not affect the OCP and stable behavior with spikes where the signal recovered the initial value but with temporary voltage spikes. Figure 7 shows an indicative OCP plot of an electrode which has undergone the voltage bias stress described in the experimental section. Here, the PRE is not affected by the 0.3 V bias (stable behavior), stable behavior with temporary spikes of 80 mV is observed for 0.6 V and unstable behavior for 0.9 V.

**Figure 8:**
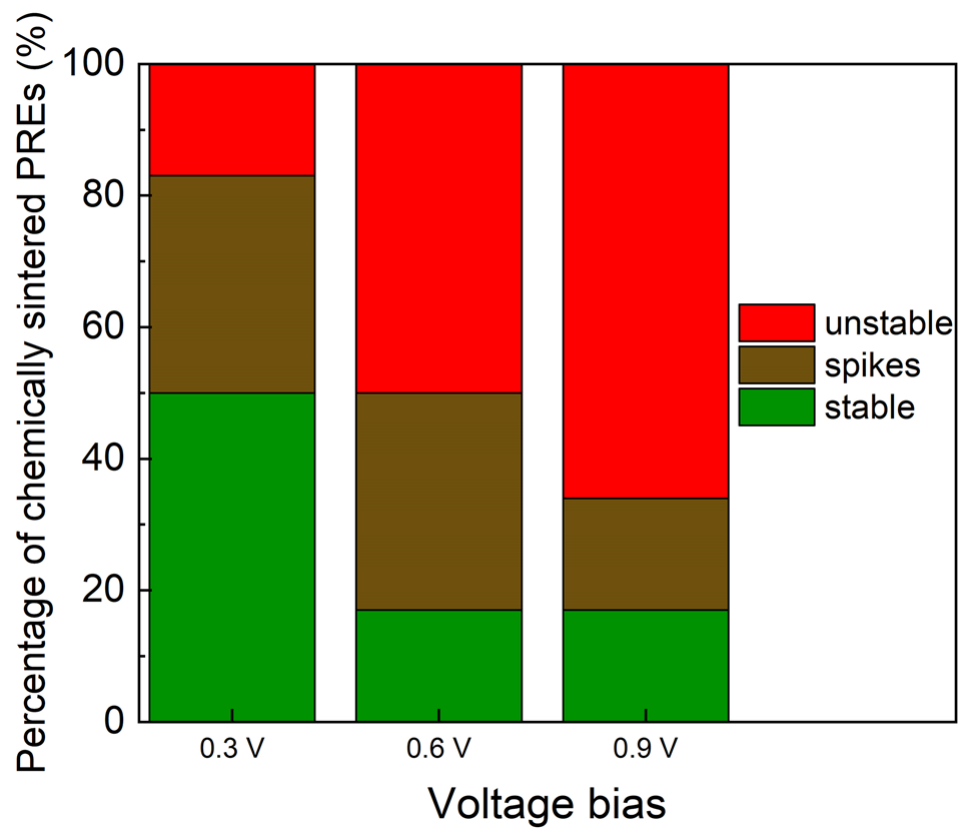
Influence of the voltage bias stress on the stability of the chemically sintered PREs with the corresponding percentages of the tested samples.

As Fig. 8 reveals, 17% of the chemically sintered PREs were unstable even after the lowest tested stress voltage bias (0.3 V) whereas 17% were unaffected by any tested voltage bias. Finally, 50% were stable after the 0.3 V bias and 17% were stable after the 0.6 V bias. These findings prove for the first time through systematic reference electrode stress analysis that the reference microelectrode material aging under voltage bias stress has to be taken into serious consideration when designing integrated electrochemical biosensors and diagnostic microsystems, as the reference electrode stability will comprise a significant factor in biosensing stability and long term reliability.

### Conclusion

Commercial silver nano-particle ink was deposited on printed circuit boards using a large format inkjet-printer to evaluate its suitability for pseudo-reference electrode formation. Evaluation of the electrodes stability under static and flow condition for various post-printing treatments was performed, revealing that our chemically sintered electrodes comprise the most stable inkjet- printed reference electrodes currently available. The reference electrode aging under DC bias stress was evaluated for the first time employing repetitive On–Off bias cycles. This study highlighted that pseudo-reference electrode electrical stress is a significant factor in electrochemical biosensor instability over long-term measurements, nonetheless largely ignored until now by researchers as a source of potential error. This work proved the potential for ultra-stable inkjet-printed pseudo-reference electrodes on PCB electrochemical biosensing platforms, promising even more stable devices under repetitive bias cycles in a real-life implementation.

## Supplementary information

Supplementary Information 1
