# Supplementary material for: Ultra stable, inkjet-printed pseudo reference electrodes for lab-on-chip integrated electrochemical biosensors
Source: Sci Rep. 2020 Oct 13;10:17152. doi: 10.1038/s41598-020-74340-1 (PMC7554035; doi:10.1038/s41598-020-74340-1)
Supplement: Supplementary file 1 — Supplementary Information 1 [file 41598_2020_74340_MOESM1_ESM.doc]

Supporting Information

**Ultra Stable, Inkjet-Printed Pseudo Reference Electrodes for Lab-on-Chip Integrated Electrochemical Biosensors**

Sotirios Papamatthaiou^1^*, Uros Zupancic^1^, Curran Kalha^2^, Anna Regoutz^2^, Pedro Estrela^1^, Despina Moschou^1^

^1^Centre for Biosensors, Bioelectronics and Biodevices (C3Bio) and Department of Electronic & Electrical Engineering, University of Bath Bath, BA2 7AY, United Kingdom

^2^Department of Chemistry, University College London, London, WC1H 0AJ, United Kingdom

* E-mail: spapamat@bath.ac.uk


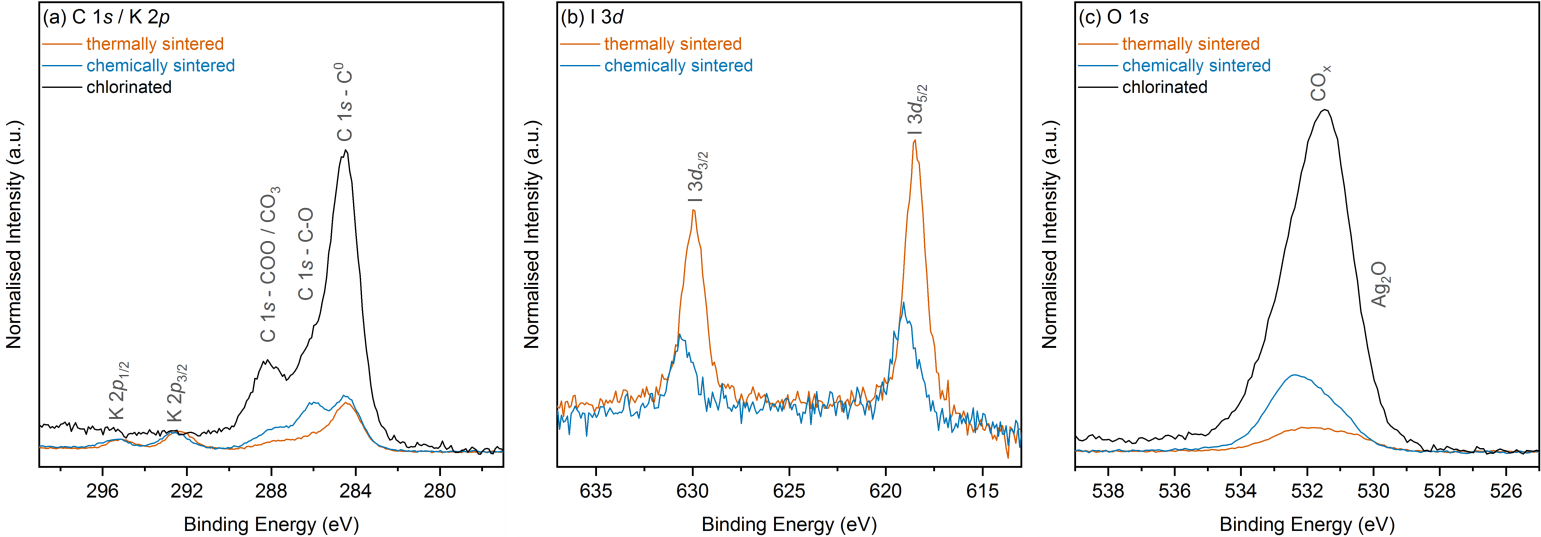


**Figure S1.** XP core level spectra of thermally and chemically sintered as well as chlorinated electrodes, including a) C 1*s*/ K 2*p*, b) I 3*d* and c) O 1*s*.


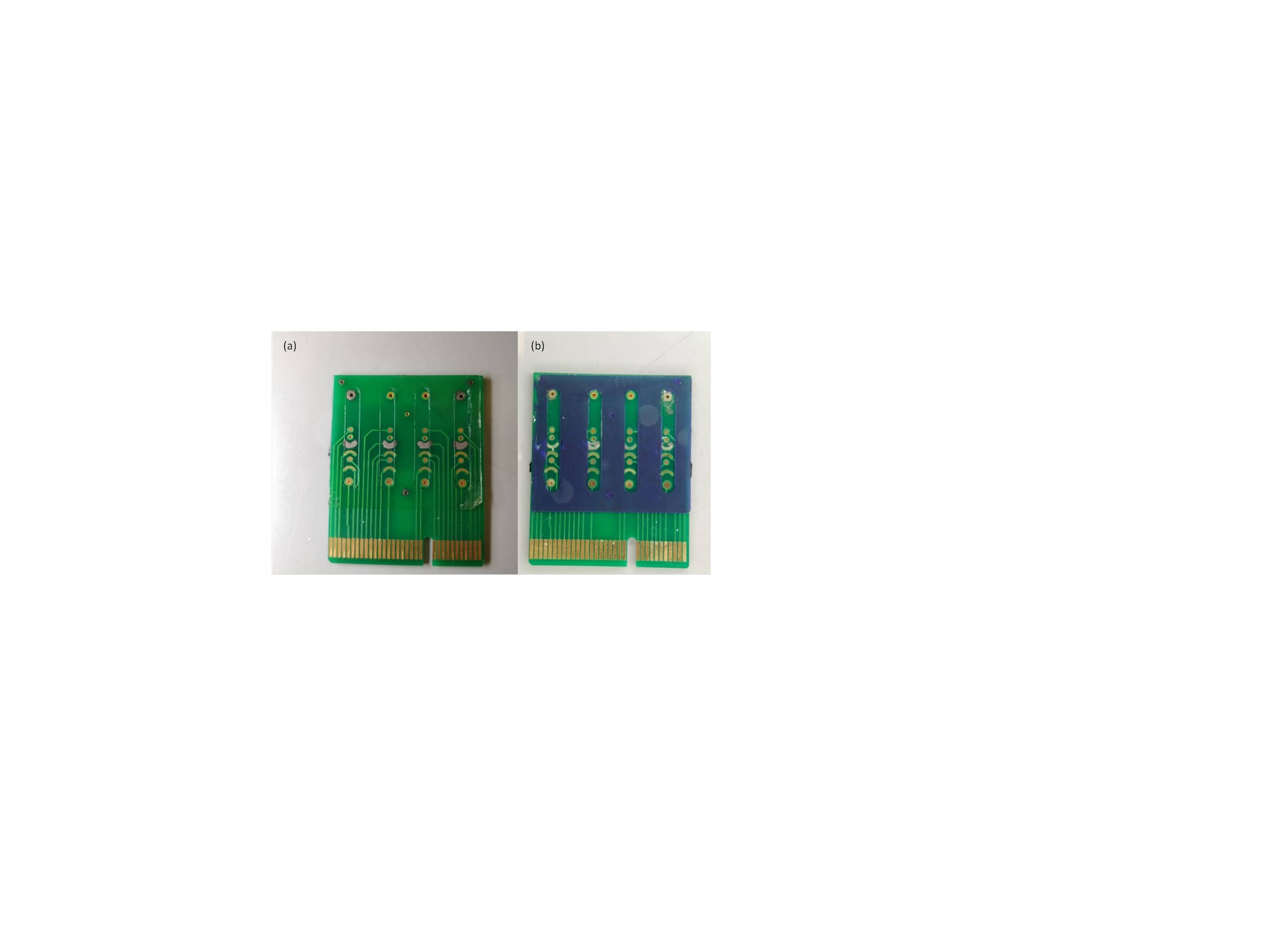


**Figure S2**. Photographs of inkjet-printed, a) chemically sintered and b) chlorinated silver reference electrodes taken after 24 h of continuous buffer flow.

**Table S1.** Total relative atomic ratios (in rel. at.%) determined from the overall peak area of the core level spectra from XPS characterisation of the electrode surfaces.

| **Sample** | **Ag 3*d*_5/2_** | **Cl 2*p*** | **K 2*p*_3/2_** | **I 3*d*_5/2_** | **C 1*s*** | **O 1*s*** |
| --- | --- | --- | --- | --- | --- | --- |
| Thermally sintered | 24.2 | 0.9 | 3.8 | 1.2 | 49.8 | 20.1 |
| Chemically sintered | 14.2 | 3.0 | 1.9 | 0.4 | 48.9 | 31.7 |
| Chlorinated | 2.3 | 7.3 | 0.1 | >0.1 | 53.8 | 36.4 |
